# Bio-objects’ political capacity: a research agenda

**DOI:** 10.3325/cmj.2013.54.206

**Published:** 2013-04

**Authors:** Pieter Maeseele, Kim Hendrickx, Vincenzo Pavone, Ine Van Hoyweghen

**Affiliations:** 1 Faculty of Political and Social Sciences, University of Antwerp, Antwerp, Belgium *pieter.maeseele@ua.ac.be*; 2 Spiral, University of Liège, Liège, Belgium; 3 Instituto de Políticas y Bienes Públicos, Consejo Superior de Investigaciones Científicas, Madrid, Spain; 4 Faculty of Social Sciences, University of Leuven, Leuven, Belgium

## Abstract

This article explores the merits of foregrounding the dichotomy of politicization vs de-politicization for our understanding of bio-objects in order to study their production, circulation, and governance in European societies. By asking how bio-objects are configured in science, policy, public, and media discourses and practices, we focus on the role of socio-technical configurations in generating political relations. The bio-object thereby serves as an entry point to approach and conceptualize “the political” in an innovative way.

Drawing from our previous work, which uses the concepts of de-politicization and (re-)politicization, this paper puts forward a research agenda for studying the political relations generated by specific socio-technical configurations of bio-objects.

## (De)politicization and democracy

The dichotomy of politicization vs de-politicization originated as a response to the idea of the arrival of a “post-ideological” era when the victory of capitalism and liberal democracy over its counterparts put forward the belief in a universal rational consensus, with experts reconciling conflicting interests and values through impartial procedures and technical knowledge (1-3). A specific school of political philosophers (4-7) however have criticized this conceptualization as embodying not so much a “post-ideological,” but “post-political” and “post-democratic” condition. This implies that the essence of democratic politics, ie, the confrontation of hegemonic political projects, is abandoned in favor of a “de-politicized” technocratic management of social, economic, and ecological matters within the framework of an “inevitable” hegemonic neoliberal project and global market forces. In this process of “de-politicization,” “the political” is transformed from a matter of ideological contestation to a matter of administration. Decision-making is no longer a question of political position but of expert knowledge, thereby foreclosing a democratic struggle between alternative courses of action beyond the existing socio-political status-quo, and replacing it with technocratic decision-making and/or market forces. In this post-political condition, anyone who disagrees with this “consensus” is turned into a fundamentalist, traditionalist, or blind radical, through a moralization and rationalization of politics. This implies that the construction of the we/they opposition in political categories constitutive of democratic politics, is, respectively, replaced by the moral categories of “good” vs “evil,” or neutralized by striving for a consensus reached by “rational” argumentation between “rational” experts (5,8). Consequently, the dichotomy politicization vs de-politicization serves as a framework for revealing strategies of in- and exclusion, and more specifically, how processes of de-politicization separate legitimate actors, demands, discourses, and practices from illegitimate actors, demands, discourses, and practices, excluding the latter from democratic debate. By addressing how specific “political relations” follow from the articulation/disarticulation of discourses and practices associated with the creation/abolition of selected institutions, this framework is primarily concerned with the hegemonic constitution of society. And more specifically, the role of power relations in the construction of particular forms of objectivity. Not only does this framework negate a view of politics as a set of supposedly technical moves and neutral procedures, it also reveals this view as a particular strategy to de-politicize and naturalize particular interests and power relations, exactly by disclosing the political nature and implications of supposedly technical moves and neutral procedures (9). Recently, previous work of the authors of this paper has exposed the political implications of socio-technical configurations of bio-objects in science, policy, public and media discourses, and practices by focusing on processes of politicization and de-politicization.

## (De)politicization in public and media discourses on genetically manipulated (GM) food

In the field of media sociology, recent framing and critical discourse analysis research on public and media discourses about GM food has demonstrated the extent to which this “bio-object” is the subject of struggles between processes of politicization and processes of de-politicization (10,11): de-politicizing discourses underpin the common problem definition with which GM food is approached in public and media discourses, successfully linking neoliberal discourses to discourses of scientism and supporting the idea of the marketing of GM food as a *normal* element of an *inevitable*, *natural* scientific and economic development serving the public interest. As a consequence, democratic control (either on science or on markets) can be interpreted as disruptive and counterproductive. This configuration naturalizes the development of GM food (and the associated technology) as scientific and economic progress, while objectifiying the interests of its developers and advocates as public interests. The consequences of these processes of naturalization and objectification are 2-fold: (A) any disruption of this natural process (eg, political debate, regulation, direct action) is interpreted as damaging to scientific and economic progress, and consequently the public interest, and (B) it can only be explained by factors external to the technology or its developers (eg, flawed science, flawed scientists, sensational media, flawed PR-efforts, an emotional, fearful, ignorant public, etc) and by stigmatizing challengers (eg, as anti-science radicals, fundamentalists, green terrorists, neo-luddites, etc). The main discursive practice emanating from this configuration is the exclusive allocation of any epistemic authority (in terms of an unproblematic notion of scientific consensus and discourses of “sound science”) to the developers/advocates of this bio-object. In so doing, unacknowledged value-laden assumptions and material interests in the development of GM food are isolated, and the “sound science” of its developers is distinguished from the epistemically-vacuous concerns of whoever resists their risk definitions. The struggle is thus shifted from a politico-ideological debate about alternative technological futures to dichotomies such as science vs politics/ideology/fear, rationality vs emotionality, sound science vs junk science, etc – with the effect of delegitimizing any space for democratic debate about alternative technological futures (10,11). This particular GMO configuration therefore mainly works in the service of evacuating a democratic debate between competing, yet legitimate actors, with competing, yet legitimate, risk definitions. In addition democratic debate or political intervention is delegitimized as not in the public interest.

On the other hand, discourses promoted mainly by social movements/NGOs were found to start from other underlying, implicit problem definitions which regard technological and economic developments not as “inevitable” or “natural” processes but as contested processes, whose direction is steered by specific agents with specific values, interests, and aspirations. The discursive practices emanating from these alternative problem definitions aimed at revealing the competing sets of assumptions, values, and interests, underlying opposing responses to scientific uncertainty (such as corporate control, financial interests, technological progressivism, a large-scale, industrialized, energy- and capital-intensive agriculture, etc.) Therefore, a discursive space for approaching the development of GM food as a conflict between alternative technological (and economic) futures was created, making it the object of public accountability, democratic debate, and political regulation. Social movements/NGOs were found to respond to the widespread de-politicizing discourses with (re-)politicizing discourses by framing the GM food controversy as a struggle between conflicting – yet legitimately considered – alternative futures proposed by conflicting – yet legitimately considered – actors. They promoted democratic debate in the face of heavy artillery by science organizations and corporate associations who in the promotion of specific products (and their associated technology) preferred to frame GM food as a predefined matter belonging to the realm of technocratic decision-making and/or “free market” forces.

For instance, Syngenta’s campaign to promote the use of GM crops to tackle water scarcity illustrates this point. In their campaign, water scarcity is framed as a technical issue, which paves the way for a technological solution (ie, GM crops). This approach obscured not only the whole array of social, economic, and political factors which have resulted in the overuse and pollution of water, but also the role played by the very innovation regime of which Syngenta’ s strategy is a part. Syngenta´s campaign is an example of how the framing of social, economic, and political problems in terms of technical questions aims at delegating essentially political decisions to expert committees, in order to divert responsibility from political actors to techno-scientific networks, while denying the relevant normative/political dimensions (12-14).

## From evidence-bases to re-politicization in insurance practices and food safety

Literature in the field of Science & Technology Studies (STS) has demonstrated the extent to which the promotion of a scientific technocratic approach in public policy serves as a de-politicizing strategy. This is visible in terms of the rise of experts, testing and monitoring activities, as well as the installation of guidelines and quality standards. The idea is created that scientific, “politically- neutral,” facts serve as a foundation for compromises and consensus in political conflict. Scientific expertise and calculative technologies are used as anti-political devices in order to reduce the space of the political and de-politicize potentially controversial issues (15-18). However, recent research on insurance practices and food safety has illustrated how recurrent processes of de-politicization, instead of closing down the political, may become a source for *re-politicization.*

A widespread tendency to tackle “rationally” political issues such as rising health care costs or discriminatory practices in insurance results in the construction of “evidence-bases.” For example, Van Hoyweghen and Horstman (19) have investigated the “insurance talk” of risks, medical risk profiles, statistics, and “evidence-based underwriting.” They demonstrate how the European insurance industry, in reaction to the public debate on the use of genetics in insurance, has explicitly reinforced its principle of “fair discrimination” based on “sound” actuarial science. Strategies of “Evidence-Based Rating” (EBR) have been deployed in order to stress the scientific base of medical risk selection. These calculation devices are deployed to de-politicize contentious issues such as the use of genetic information in insurance. This politics of calculation, instead of closing down the political, becomes a source of re- politicization (20). The strategy of evidence-based underwriting was found to create particular expectations, making uncertainty in insurance underwriting more explicit than previously. Effectively, thus, the turns toward evidence-based underwriting standards and the intensification of calculation in insurance provokes new sources of politicization. In other words, insurance generates its own forms of failure and resistance. The intensification of medico-actuarial investments makes it vulnerable to further interrogation and to possible politicization. While the intentions of evidence-based underwriting strategies are to de-politicize the genetics issue, its effects appear to be political (again). The evidence-based machinery intensifies insurance markets as scenes for political conflict. Insurance is a “regime of inclusion-exclusion” (21) *par excellence* and the recent turn toward measurement (and explicit “evidence-based” calculation) is likely to amplify this dimension even more.

Another illustrating example proceeds from the case of bovine spongiform encephalopathy (BSE). In relation to food safety, the BSE or “mad cow disease” crisis was termed a *crisis* in the EU, and not just a (serious) problem to deal with, because food safety explicitly became the site of intense political conflict. Confusion abounded as to who decided what within the European Commission. The latter was criticized of being an obscure body where political interests and technical expertise (mainly British veterinarians) coincided. Scientific judgment about the risks of contamination was deemed to be contaminated *itself* by political interests. The EC had lost credibility and, above all, trust. To *restore* citizen's and consumer's trust, it was decided to reconfigure the area of food policy. The European Food Safety Authority (EFSA) was created to centralize scientific expertise in Parma, *removing it physically* from decision-making bodies in Brussels. Food policy thus became a de-politicized mode of public intervention, where decision-making relied upon information obtained *elsewhere* through the scientific practice of risk assessment. Indeed, it was the principle of risk analysis, as originally proposed by the US National Research Council in the 1980s, that inspired European policy-makers to reform the area of food policy and create EFSA as a separate scientific agency (22). In an attempt to de-politicize the politics of risk, the principle of Risk Analysis separates risk assessment from risk management, the latter depending on the former to provide a politically “neutral” evidence-base for subsequent decision-making. However, EFSA immediately became the target of critique, and new controversies proliferated again. The separation of scientific risk assessment from political risk management has not been able to prevent political conflicts around issues like GMOs, nutrition, and health claims, but it rather *displaced* and changed existing conflicts (23). For example, when EFSA was charged to scientifically assess health claims on food products – a matter that was subject to rather loose national regulations before – the agency and its assessment scheme became an obligatory reference point, not only for industry, but indirectly also for academic scientists researching the health benefits of specific food compounds. A debate is currently going on about the scientific evidence needed to make such health claims about a food product. Implementing a particular version of evidence-based medicine (EBM), EFSA requires data from randomized, placebo-controlled clinical trials on *healthy populations*. Evidence on clinical endpoints related to *disease* is however not allowed, as the product would then be considered a “drug” and not a “food product.” This has triggered reactions and the formation of new groups defending *their* science, and not necessarily Science (with a capital) in general. These groups are not just new “stakeholders” that claim to be concerned by a given, stable object, but *contesters,* calling into question the meaning of “scientific proof” or the divide between food and medicine. They actively make propositions to enable food ingredients to develop new therapeutic relations and contest the techno-legal identity of the food, which becomes a bio-object in the configuration of European food policy.

## Toward a research agenda: translations of the political

While de-politicization and (re-)politicization have been variously approached in the examples we discussed, they have in common that politicization represents the process of opening-out issues to disagreement, conflict, and alternative framings (of socio-political relations). The role of technocratic, administrative, managerial, expert-led, and consensus-seeking approaches and procedures in foreclosing processes of politicization is represented by the process of de-politicization. In this latter process, the rise of experts, testing and monitoring activities, the installation of guidelines and quality standards, the construction of evidence-bases as well as calculation devices, are put forward as supposedly technical moves and neutral procedures, promoted by the cultural authority of science, scientism, and metrication. By isolating unacknowledged value-laden assumptions and material interests in their articulation and/or institution, these practices and discourses serve as de-politicizing strategies, which aim at the naturalization of particular values, interests, and (unequal) socio-political relations. In these processes, the *rational* and *moral* “sound science” of responsible actors and demands is distinguished from the *radical* epistemically-vacuous concerns of irresponsible actors and demands. This shifts the site of struggle from politico-ideological conflict about alternative courses of action to dichotomies such as (sound) science vs politics/ideology/fear/emotions/junk science. It evacuates a democratic debate between competing, yet legitimate actors with competing, yet legitimate demands in favor of technocratic decision-making or “free market” forces (reproducing the socio-political status-quo). The proliferation of “evidence-bases” in different professional milieus such as medicine, nutrition, and insurance practices points at the instauration of axes that discriminate between what is valued and what is not in these practices, in other words, between what is recognized as legitimate and what is recognized as illegitimate. However, these examples have simultaneously demonstrated how in these socio-technical configurations the political is never closed down, since these configurations subsequently become a source of re- politicization.

From a perspective of democratic politics, the identification of processes of de- and re-politicization constitutes an important research agenda for scholars working on bio-objects. We call this agenda, “translations of the political,” since these processes *translate the political* differently: processes of *de-politicization* involve the construction of specific and new bodies of knowledge such as “evidence-bases,” which are not directly based on scientific research results, but rather on ways to *categorize* research results in such a manner that political decisions can be made and legitimized. De-politicization thus means making the political invisible for political purposes. The contingent nature of such categorization practices becomes apparent through contestation and alternative categorizations in what we have called processes of *re-politicization.* In so doing, we align ourselves with recent work of science and technology studies (STS) researchers who have sought to bring the question of politics in STS research (24,25). Other work in this field has drawn on neo-Foucauldian strands of social theory, which stress the importance of socio-historical formations like “neo-liberal governmentality” (26,27), post-marxist theories like Gramsci’s concept of hegemony (28), or Marx’s concept of mode of production (29-31). The “political turn” we are calling for is to study discourses and practices as intertwined elements of the specific socio-technical configurations in which they function. The analytical challenge is to understand these context-specific arrangements in terms of in/excluded, il/legitimate ideas and dreams, facts, spokespersons, (bio-)objects, professional groups, and forms of knowledge, and to analyze their mutual *effects* in generating the political.

Moreover, bio-objects, as *altered or new biological entities,* are particularly hard to categorize. The proliferation of genes, proteins, embryonic cells, GMOs, and so on, as “living entities” (32) raises multiple problems of categorization, standardization, and framing. The intricacies in coming to terms with “living entities” in legal lawmaking (eg, GMO regulation, patent law and embryo law), and in suppressing natural complexity with the mechanisms of laws, have been documented (33-35). This difficulty in “taming” life itself re-generates political controversy and the emergence of concerned groups. With commercial interests in mind, bio-objects raise specific *stakes* when it comes to their definition, their categorization, and their conditions of diffusion (as in European regulation for example). A fundamental issue for this research agenda is the empirical exploration of whether bio-objects, as biological matter “out of place,” generate *specific kinds* of political relations. As pinning down bio-objects in a single legal or technical definition is extremely difficult, an important question becomes: To what extent can a bio-object be said to socially exist, and maintain a fixed identity, given that the qualification of the object *is precisely what is at stake* in processes of de- and re-politicization? Essentially, we invite our colleagues to explore *bio-objects* and their capacity to generate political relations, in addition to new and conflicting definitions of what a given bio-object is. Because of the prevailing de-politicization of debates such as GM crops and food in terms of “facts and figures,” we hold that the subsequent contestations and re-politicizations should not be regarded as obstacles to progress, but as conditions for an alternative approach to progress: The question is not what kind of *technological solutions we “need*,*”* according to the latest prospective study, but what kind of *society we want*, according to the bet that different dreams can be made to co-exist.

Acknowledgments The authors thank the stimulating environment of the *ISCH COST Action IS1001*: Bio-objects and their boundaries: governing matters at the intersection of society, politics and science, and more specifically Working Group 3: The emergence of new kinds of social and economic relations prompted by processes of “bio-objectification.”
